# Comparison of Electroacupuncture in Restrained and Unrestrained Rat Models

**DOI:** 10.1155/2013/404956

**Published:** 2013-05-12

**Authors:** Haolin Zhang, Xiaolong Chen, Chan Zhang, Ruixin Zhang, Lixing Lao, You Wan, Ming Yi

**Affiliations:** ^1^Neuroscience Research Institute & Department of Neurobiology, School of Basic Medical Sciences, Peking University, 38 Xueyuan Road, Beijing 100191, China; ^2^Center for Integrative Medicine, School of Medicine, University of Maryland, 685 W. Baltimore Street, MSTF Rm 8-22, Baltimore, MD 21201, USA; ^3^Key Laboratory for Neuroscience, Ministry of Education/National Health and Family Planning Commission, Peking University, 38 Xueyuan Road, Beijing 100191, China

## Abstract

Acupuncture and electroacupuncture (EA) are widely used to treat a variety of diseases including pain. In preclinical research, EA is usually applied by inserting acupuncture needles into the hindlimbs of rats restrained in small tubes or bags. This restrained model of EA not only causes stress-like behaviors but also is limited in stimulating locations and intensities. In 2004, a novel, unrestrained model of EA was introduced. However, these two EA methods have never been directly compared regarding their analgesic effects and other features such as stress. In the present study, we reported similar analgesic effects between restrained and unrestrained EA in rats of acute inflammatory pain induced by intraplantar injection of CFA. In addition, rats receiving unrestrained EA showed less significant stress-like behaviors and tolerated higher current intensity. These advantages suggest that this unrestrained EA method can replace the traditional restrained procedure with similar analgesic effects and allow for more choices of stimulating intensities and locations.

## 1. Introduction

Acupuncture and electroacupuncture (EA) can effectively treat a variety of diseases such as pain and nausea. Animals, especially rodents, are now widely used in preclinical research on the neural mechanisms of EA. In the traditional EA model, conscious rodents are restrained in small tubes or bags, with acupuncture needles inserted into their hindlimbs [1, 2]. This restrained EA method had been suggested to cause stress [1], and its analgesic effects might hardly be differentiated from stress-induced analgesia (SIA) [3]. In addition, accumulating evidence suggests that different stimulating parameters, such as current intensity, frequency, and location, all significantly affect the analgesic effects of EA [2, 4–9]. The restrained EA model is limited in stimulating locations and intensities, that only acupoints on hindlimbs can be stimulated with relatively low intensities to avoid stress-like responses such as vocalizations and muscle twitches. Finally, *in vivo* electrophysiological techniques in consciously behaving rodents are more and more widely used in neuroscience research, including pain studies [10, 11]. The presence of restraining tubes or bags restricts their application in EA research. In 2004, Lao et al. [12] introduced a novel, unrestrained method of EA stimulation. This model does not require restraining tubes or bags and allows for more choices of acupoints, for example, those on the back. However, the analgesic effects, intensity tolerance, and stress-like behaviors have never been directly compared between these two EA methods. The present study was designed to answer these questions.

## 2. Material and Methods

### 2.1. Animals

**Figure 1:**
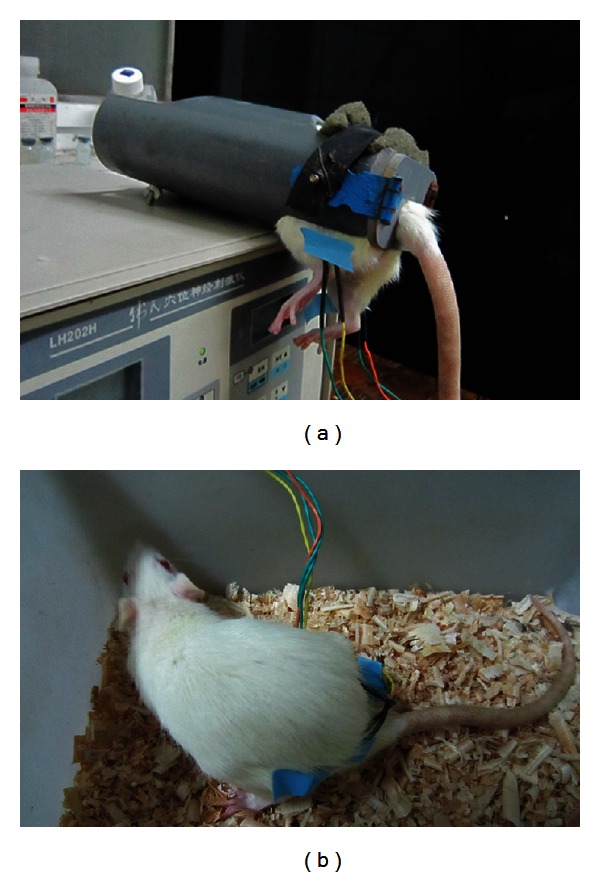
Photographs showing the restrained EA (a) and the unrestrained EA (b) procedures.

Male Spraque-Dawly rats were provided by the Department of Experimental Animal Sciences, Peking University Health Science Center. They were housed 4–6 per cage with the temperature maintained at 22 ± 1°C and kept under a natural light/dark cycle. Food and water were available ad libitum. Rats were handled daily for minimally three days before any experiments. During this period and regardless of their grouping, rats were also habituated to EA tubes, by 10 min daily restraint (Figure 1(a) but without acupuncture needles), and to EA wires, by 10 min daily free exploration in small cages (Figure 1(b) with acupuncture needles taped but not inserted onto their bodies). All animal experimental procedures were conducted in accordance with the guidelines of the International Association for the Study of Pain and were approved by the Animal Care and Use Committee of Peking University.

### 2.2. The CFA Model of Inflammatory Pain, EA, and Thermal Hyperalgesia Measurement

The rat model of acute inflammatory pain was established by intraplantar injection of 0.1 mL Complete Freund's Adjuvant (CFA, Sigma, suspended in an 1 : 1 oil/saline emulsion, 0.1 mL, and 50 *µ*g Mycobacterium tuberculosis) into the left paw [12]. These inflamed rats were then randomly divided into three groups. Rats in the restrained EA group (*n* = 12) and in the unrestrained EA group (*n* = 12) received EA treatment. For restrained EA, rats were restrained in plastic tubes with hindlimbs extending out through two holes (Figure 1(a)). Bilateral GB30 (Huantiao, located at the junction of the lateral 1/3 and medial 2/3 of the distance between the greater trochanter and the hiatus of the sacrum) [12] were stimulated with square waves of 0.2 ms in pulse width and 100 Hz in frequency from an Han's Acupoint Nerve Stimulator (HANS, LH series, manufactured in Peking University). Their intensities were increased in a stepwise manner at 1.0-1.5-2.0 mA, each lasting for 10 min [2]. For unrestrained EA, one investigator gently held the animal, while another swiftly inserted acupuncture needles into bilateral GB30. The needles were stabilized with adhesive tape. The procedure typically lasted less than 20 seconds and caused little distress. These rats were then released in a small cage to receive EA stimulation of the same intensities (Figure 1(b)). If the needles dropped during EA, they were inserted again, and the procedure took a few seconds. To obtain strong analgesic effects, each rat received two EA sessions, one immediately after CFA injection and one 2 hours after injection as previously described [12]. Needles were pulled out, and rats were released into their home cages between these two sessions. Rats in the control group (*n* = 10) were either restrained in tubes as in the restrained EA group (*n* = 5) or freed in a cage as in the unrestrained EA group (*n* = 5) but did no receive EA treatment. No differences of paw withdrawal latency (PWL) changes were detected between these two manipulations at any time points so they were pooled into the control group (restrained controls versus unrestrained controls: 2.5 h: −16.4 ± 3.3 versus −16.9 ± 3.7; 5 h: −17.2 ± 2.3 versus −17.8 ± 2.8; 24 h: −17.3 ± 2.7 versus −19.9 ± 4.1, seconds, *P* > 0.05 for all time points).

The PWL was measured in a blind manner before CFA injection and 2.5 hours, 5 hours, and 24 hours after injection. The rat was placed under a clear plastic chamber on a glass surface. A high-intensity beam (IITC, Woodland Hills, CA; setting = 20%, *≈*45 W) was applied onto the plantar surface of the left hind paw from underneath the glass floor. The PWL was measured to the nearest 0.1 s when the rat withdrew its hind paw from the radiant heat stimulus and mean PWL was calculated by averaging the latencies of three tests with 5 min intervals between each test. With this intensity, naive rats showed an average PWL of approximately 20 seconds. Thirty seconds was used as the cutoff time to avoid plantar injuries. The PWL before CFA injection was taken as the baseline. Changes of PWL after CFA injection were calculated by subtracting the baseline PWL from the measured PWL.

### 2.3. Current Intensity Tolerance and Stress-Like Behaviors

We next tested the highest current intensity that could be tolerated by rats with restrained and unrestrained EA. Naive rats received stimulation in bilateral GB30 as above. The current intensity was increased from 1.0 mA at 0.5 mA steps (60 seconds per step) to the level that caused hindlimb flinches or audible vocalizations and maintained at this level. This procedure lasted 5 minutes so the maximal current intensity was 3.5 mA. The highest current intensity that did not cause any hindlimb flinches or vocalizations was defined as the current intensity tolerance. Five other parameters were measured during this 5 min period: number of hindlimb flinches, number of urination, number of audible vocalizations, duration of audible vocalizations, and number of fecal boli.

## 3. Results

**Figure 2:**
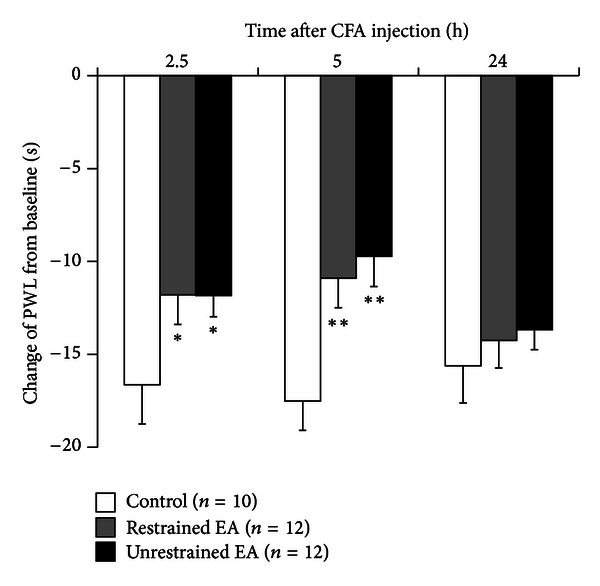
Restrained and unrestrained EA produced significant and similar analgesic effects 2.5 and 5 but not 24 hours after intraplantar CFA injection, indicated by decreased changes of PWL from preinjection baseline. *P < 0.05, **P < 0.01 compared with the control group, ANOVA with Dunnett's post hoc tests.

After subcutaneous CFA injection into the plantar surface of left hind paws, rats developed thermal hyperalgesia, indicated by a sharp decrease of PWL from thermal stimulation which lasted for over 24 hours (Figure 2). Restrained and unrestrained EA produced significant and similar analgesic effects 2.5 and 5 but not 24 hours after injection, indicated by smaller PWL changes from preinjection baselines (2.5 h: *F* = 4.15, both *P* < 0.05; 5 h: *F* = 5.06, both *P* < 0.01; 24 h: *F* = 0.36, both *P* > 0.05 compared with the control group, ANOVA with Dunnett's post hoc test. Figure 2). However, there were no differences between the two EA methods (*P* > 0.05, ANOVA with Dunnett's post hoc test. Figure 2). 

**Figure 3:**
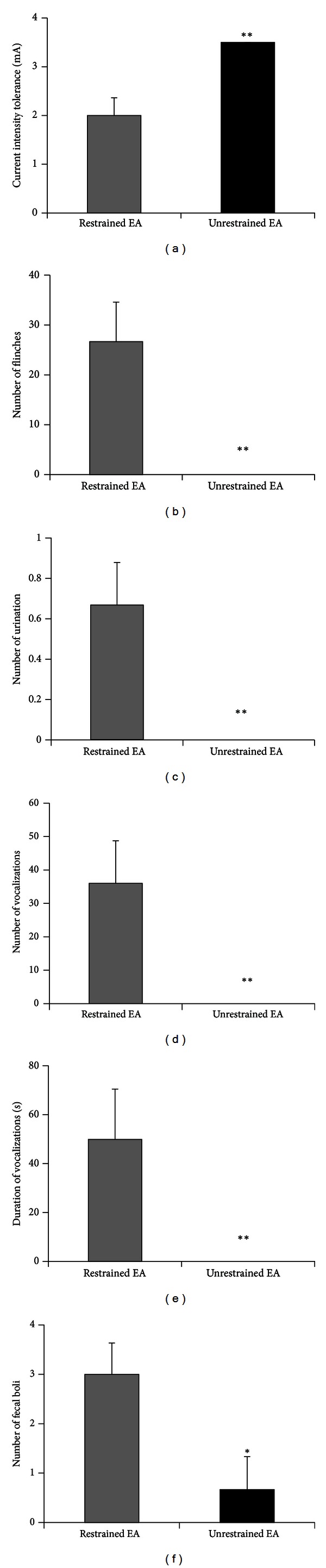
Current intensity tolerance and stress-like behaviors during restrained and unrestrained EA. (a) Rats with unrestrained EA could tolerant significantly higher current intensity than rats with restrained EA. Rats with unrestrained EA showed significantly fewer hindlimb flinches (b), number of urination (c), number of audible vocalization (d), duration of audible vocalization (e), and number of fecal boli (f) during EA procedures. *P < 0.05, **P < 0.01, and Students *t*-test.

In both human volunteers [5, 6] and anaesthetized rodents [9], there is evidence that EA of different intensities exerts analgesic effects through distinct mechanisms. However, it is hard to perform such experiments in awake animals with the restrained EA method, since rats start to show stress-like behaviors, indicated by strong hindlimb flinches, vocalization, and fecal boli, when the current intensity is increased to higher than 2 mA (Figure 3). Using the unrestrained methods, we noticed that the intensity tolerance was significantly higher (*P* < 0.01, Students *t*-test). Indeed, all rats receiving unrestrained EA could tolerate up to 3.5 mA currents with little indication of stress (Figure 3(a)). The number of hindlimb flinches (*P* < 0.01, Students *t*-test), the number of urination (*P* < 0.01, Students *t*-test), the number of audible vocalizations (*P* < 0.01, Students *t*-test), the duration of audible vocalizations (*P* < 0.01, Students *t*-test), and the number of fecal boli (*P* < 0.05, Students *t* test) were all lower in the unrestrained EA group than in the restrained EA group (Figures 3(b)–3(f)). 

## 4. Discussion

The neurobiological mechanisms of EA analgesia are intensively studied worldwide. It is generally accepted that EA promotes the release of analgesic substances such as opioids [4]. However, more recent work indicated distinct mechanisms of EA analgesia between different stimulating frequencies [4, 7], durations [7, 8], intensities [5, 6, 9], locations [2], and subject conditions (healthy versus pathological states) [13]. Thus, various experimental designs and techniques should be applied in combination towards a comprehensive elucidation of EA mechanisms. Rodents are traditionally placed into special tubes or bags for EA stimulation [1]. This method has obvious limitations. Firstly, only a limited numbers of acupoints, mainly those in the hindlimb, could be stimulated. Acupoint specificity has raised lots of attention, not only that different acupoints show distinct analgesic effects under pathological conditions but also that the underlying mechanisms vary as well [2]. The presence of restraining bags or tubes prevents adequate animal experiments on this issue. It also restricts the application of *in vivo* electrophysiological recording in consciously behaving rodents [10, 11] in EA research, since the animal's head is usually inaccessible under such conditions. Finally and most importantly, animals sometimes show stress-like behaviors such as vocalization and hindlimb flinches when restrained in small containers. Such behaviors cause intolerance to high current intensities and confuse mechanisms of EA analgesia with those of stress-induced analgesia (SIA) [3].

In the present study, we tested a new unrestrained EA method first described by Lao et al. [12]. We found that without restraint, rats tolerated a much higher current intensity with few signs of stress-like behaviors. Our experiment in CFA-induced inflammatory pain revealed similar analgesic effects between the restrained and the unrestrained EA methods. These data suggest that the unrestrained EA method could fully replace the restrained model and allow the application of a broader range of stimulating intensities and locations as well as *in vivo* recording in conscious rodents. The presence of stress-like behaviors during restrained EA requested differentiation between EA analgesia and SIA. In clinical situations, acupuncture or EA may cause deqi sensations characterized by aching, pressure, heaviness, and numbness but not negative emotions such as sharp pain [14]. Emotional stress could be easily avoided in humans with sufficient verbal communications and habituation. In rodents, however, stress-like responses frequently occur during EA stimulation under restraint. In the present study, for example, despite the intensive handling and habituation, rats still showed stress-like behaviors such as hindlimb flinches and vocalization in the restraint tube when the current was increased to ~2 mA. In an early study, Wan et al. [1] showed that restraining mice in clothing holders alone was sufficient to produce mild anti-nociception, but EA stimulation showed an additional analgesic effect. Thus, restraint becomes a confounding factor in evaluating the real analgesic effects of EA and explaining its mechanisms. In our CFA experiment, we tried to eliminate stress by intensive handling and habituation as well as by using relatively low current intensity (1-1.5-2 mA). Under this condition, little evidence of stress was observed during the procedure. Despite these manipulations, the analgesia effects in the restrained EA model may still be partially caused by SIA. A more direct way to reflect stress level is to measure blood biochemical indexes. Our previous study [13] showed that unrestrained EA increased blood corticosterone levels in rats with intraplantar CFA injection but not in naïve rats without inflammatory pain. Adrenalectomy blocked EA-produced anti-edema, but not EA anti-hyperalgesia [13]. These data suggest distinct working mechanisms of EA in healthy subjects and in those with pathologies, and that changes of hormone levels could be independent from pain behaviors. In the present preliminary study, we observed stress-like behavioral changes associated with restrained naive animals. To better elucidate how blood biochemical indexes change during EA and more importantly, how they correlate with EA induced antinociception, a more carefully designed and controlled future study is required. 

## 5. Conclusions

Overall, rats experienced less stress during the unrestrained EA procedure and tolerated higher current intensity. The absence of restraining bags or tubes also allows for more choices of acupoints and application of electrophysiological techniques that require access to the head of conscious rats. These advantages, combined with similar analgesic effects compared with the traditional restrained EA, suggest that the unrestrained EA method can replace the restrained EA procedure in future research.
